# Vision-Based Continuous Robust LOS Angle Measurement with Seamless Parameter Adaptation for Approaching a Spacecraft Component

**DOI:** 10.3390/s26051608

**Published:** 2026-03-04

**Authors:** Fei Xie, Ling Wang, Bo Wang, Jingwen Zheng, Xiang Zhang

**Affiliations:** 1 College of Electronic and Information Engineering, Nanjing University of Aeronautics and Astronautics, Nanjing 211106, China; xie_fei@nuaa.edu.cn (F.X.); wb294594@nuaa.edu.cn (B.W.); zhengjingwen@nuaa.edu.cn (J.Z.); 2 School of Mechanical Engineering, Nanjing University of Science and Technology, Nanjing 210094, China; zhxiang2002@126.com

**Keywords:** component approach, component recognition, component segmentation, line of sight (LOS) angle measurement, on-orbit servicing

## Abstract

The component-level line of sight (LOS) angle measurement of spacecraft is much desired during space rendezvous, especially for component-related operations, such as component status evaluation, component repair, etc. However, most existing methods rarely consider the component approaching scenario where a continuous, stable, real-time LOS angle measurement method for the component of interest is needed. In this paper, a continuous robust component-level LOS angle measurement method with high computational efficiency applicable to the approach of the key component is proposed. Firstly, an adaptive gamma correction method is introduced to enhance the image quality in complex and variable lighting environments. Secondly, optimized thresholding that exploits information entropy is proposed to identify the pixels that are supposed to be the target from the background. Region detection is subsequently performed to segment the target region into suspected component regions, which can account for target changes during the approach by seamless parameter adaptation. Then, solar panels are recognized and accurately segmented based on the prior knowledge of their spatial relationship with other components and unique shape features. Finally, the centers of solar panels are localized and their LOS angles are calculated. Extensive experiments are conducted to demonstrate the performance of our proposed method, including the verification of the superiority of the solar panel recognition and segmentation method using both simulated images generated by an image simulator and actual images taken by a camera in a dark-room considering the actual lighting in space, and the validation of the ability of supporting real-time component-level LOS angle measurement by ground semi-physical experiments with a guidance, navigation and control (GNC) system incorporated to simulate an on-line dynamic approach.

## 1. Introduction

With the rapid advancement of space technology, the repair, take-over, extension of service life and cleaning of damaged spacecraft are critical. As early as the 1960s, researchers began to study on-orbit servicing technologies for spacecraft [1]. The on-orbit servicing mission set refers to a series of space operations performed by humans, robots, or a combination of both, to extend the life of various spacecraft and enhance their ability to carry out tasks. Specifically, it mainly includes the module replacement, debris cleaning, material supply, on-orbit assembly, on-orbit repairing and so on [2,3,4]. To successfully carry out the aforementioned service activities, rendezvous technology is indispensable. Fehse [5] classifies rendezvous missions into five phases: (1) launch and orbit injection; (2) phasing and transfer to near target orbit; (3) far-range rendezvous operations; (4) close-range rendezvous operations; and (5) final approach. The space rendezvous mission is evolving from rapid rendezvous to high-precision autonomous navigation. Visual measurement technology has become a key technology in high-precision autonomous navigation due to its characteristics of low cost and high information redundancy.

A considerable number of the target spacecraft for on-orbit servicing are non-cooperative spacecraft; the main characteristic of non-cooperative spacecraft is the lack of active communication capabilities and predefined identifiable markers. The general framework for achieving autonomous relative navigation of non-cooperative spacecraft is to observe non-cooperative spacecraft through navigation sensors, then calculate the relative pose of the target spacecraft using the measurement data, and then determine the relative position, relative velocity, relative attitude and their rate of change between spacecraft through navigation filters. During the approach, relative navigation can be performed using range and line of sight (LOS) information [6] or only the LOS information [7]. In this study, the LOS angles, including the out-of-plane angle and the in-plane angle of a component of the target spacecraft, are measured to assist the relative navigation system in achieving mid- and close-range approaches to the target component.

According to the measurement system, navigation sensors are generally classified into active and passive types. The former uses devices such as microwave radar and Light Detection and Ranging (LiDAR) [8,9], while the latter uses optical sensors such as Charge-Coupled Device (CCD) optical cameras [10,11,12,13,14] and infrared sensors. In addition, space-based Global Navigation Satellite Systems (GNSS) are also widely used in navigation scenarios but they can only be applied to cooperative spacecraft [15]. Active sensors can provide high-accuracy pose data. However, the microwave radar is expensive, has high power consumption and is large in size, while the LiDAR has a relatively short effective measurement range. Optical cameras feature low hardware complexity, low cost, low power consumption, small size and lightweight, which can reduce research and development costs. Among them, stereo vision systems, such as a binocular camera, can acquire Three-Dimensional (3D) target information. However, the maximum measurement range of stereo vision is constrained by the baseline of the stereo configuration. The monocular camera can measure relative LOS angle information from far to close range, but it lacks depth information. Relying on a single sensor for measurement makes it difficult to meet the requirements of relative navigation for non-cooperative spacecraft. Therefore, it is essential to fuse sensors to construct a measurement system, thereby expanding the measurement range, enhancing its capability, and improving accuracy.

Non-cooperative spacecraft can be classified into two categories based on whether prior geometrical information about their shape and size is available or completely unknown [16]. In this work, this study mainly focuses on spacecraft with two symmetric solar panels; the actual length and width of the whole target are known. The approach range is assumed to be from 500 m to 10 m. Considering the approach range and the requirements of small satellites for compact payload volume, lightweight and low power consumption, a monocular camera is employed to obtain observation data, and a laser rangefinder is integrated as an auxiliary device to enable accurate distance measurement. The servicing spacecraft should maintain autonomous and high navigation accuracy of relative navigation to the critical components of the target spacecraft to ensure the successful completion of the component approach. The need for autonomy makes high-accuracy localization of the component a complex task, requiring ad hoc algorithmic and technological solutions [16].

Existing monocular vision-based pose estimation methods primarily follow two paradigms: deep learning-based approaches and geometry-based feature methods. Deep learning methods, such as the one by Wu et al. [17], take spacecraft images as input, locate the target in the image via a neural network, and then compute the relative position in space using a pinhole camera model. This method achieves the localization of the bounding box center of the target, but its accuracy is influenced by the bounding box size, particularly under varying target orientations. The end-to-end pose estimation methods based on deep learning [18,19] use images of the target spacecraft as input, and the deep neural network directly calculates the pose information. Despite their effectiveness, a key limitation of these methods is that training deep learning networks requires substantial labeled data, which is difficult to obtain for non-cooperative targets with limited prior information. Furthermore, deep learning models often lack robustness under challenging on-orbit conditions, such as drastic illumination changes, intense space radiation noise, and significant scale variations. As these complex situations are rarely fully covered in training datasets, the generalization capability of such data-driven methods is constrained in practical space applications. Additionally, although lightweight models like Convolutional Neural Networks (CNNs) demonstrate high efficiency in ground-based applications, their black-box nature inherently lacks the interpretability and determinism required for critical systems. Such methods consequently fall short of meeting the stringent reliability, determinacy, and verifiability requirements for embedded systems in astronautics. Therefore, deep learning-based methods are not employed as comparative baselines in this work. Instead, we adopt traditional image processing techniques, which offer high reliability, determinacy, and verifiability, to satisfy the rigorous engineering demands of space-based embedded applications.

Geometry-based methods estimate the relative pose without a full 3D model, which typically involves feature recognition followed by pose estimation. Common structural elements of satellites, such as the main body, antennas, solar panels with deployment mechanisms, and externally mounted brackets, offer recognizable geometric features. Features including points [20], lines, triangles [21], circles [22], or their combinations [23,24,25], have been used for component detection. However, at far range with small targets, point-based methods become unreliable. Recognition methods based on triangles and circles are typically confined to specific configurations, such as a triangular structure between the solar panels and the main body or interface rings. They generally lack the ability to recognize key operational components, which is a critical requirement for on-orbit servicing tasks, like repair, assembly, or refueling, where precise localization and tracking of component centers are essential. To address these challenges, Long et al. [24] leverage the solar panel structure and incorporate circular features with normal-direction constraints to refine pose accuracy, though the method is validated mainly at close range. Wang et al. [25] introduce a line-detection and clustering method combined with length-angle metrics to recognize quadrilateral solar panels, whereas their system still depends on You Only Look Once (YOLO) for far-range detection, inheriting data dependency and generalization limits common to deep learning. Importantly, the precise localization and continuous tracking of component centers are crucial for achieving accurate robot interaction during on-orbit operations. Robust model-free methods that maintain accuracy over medium and long distances are an important research need, particularly for non-cooperative targets with limited visual features.

The challenges of achieving component-level LOS angle measurement during the approach can be summarized into three main aspects. Firstly, as the distance decreases, the size of the target spacecraft in the image changes from small to large. The measurement method must therefore adapt to such target scale variations and maintain continuous measurement capability. Secondly, the lighting conditions in space are complex: the overall brightness changes with distance, and unidirectional sunlight causes luminance gradients across the target surface, where surfaces closer to the light source are brighter than those in the shadow. This interferes with the stable extraction of component edges and features. Thirdly, to support a fully autonomous relative navigation and closed loop control, the entire measurement process must meet the real-time processing requirements of high frame rates under limited computing resources.

Therefore, a robust vision-based method is proposed to achieve continuous LOS angles measurement for approaching a spacecraft component. Using a monocular camera system as the primary navigation sensor faces significant challenges due to its high sensitivity to variable illumination conditions of the space environment. The image preprocessing method, including adaptive gamma correction and optimized thresholding exploiting information entropy, is proposed to adapt to the light conditions in space. Region detection with seamless parameter adaptation is performed on the Region of Interest (ROI) to separate each component region; this step is the prerequisite and key technology for achieving continuous measurement. Then, the LOS angles are calculated through the angle transformation. And then, the center coordinates of components are calculated and the LOS angles are determined through the angle transformation. To handle diverse situations in the approach phase, the proposed recognition method specifically addresses anomalies caused by shadow occlusion and control deviations, enabling robust performance. The proposed method is validated using images simulated through an image simulator and captured in a dark room. The image simulator generates approach-phase imagery using actual orbital data. This process accounts for the actual lighting, the non-uniform illumination caused by the directional light source, and the addition of random noise. The dark room setup simulates the real navigation environment using a 1:70 scaled target model, a solar simulator (Tacera, Beijing, China), a three-axis turntable (Jiangyun, Beijing, China), a three-axis guideway (Shidai Chaoqun, Beijing, China) and so on. Finally, a semi-physical simulation platform integrated with the Guidance, Navigation, and Control (GNC) system is used to verify the capability of supporting real-time LOS angle measurement.

To address the above challenges, the proposed method has the following contributions:

1. To the best of our knowledge, we first propose a vision-based continuous LOS angle measurement method applicable for medium-range and close-range approaches to a component. Seamless parameter adaptation is designed to address the impact of target size changes during the approach phase, thereby enhancing the reliability of the measurement.
2. To adapt to the inconsistent brightness, an adaptive gamma correction method is employed to enhance the contrast and a local adaptive thresholding exploiting information entropy is proposed to accurately segment the target from the background.
3. By leveraging classical algorithms on the ROI and incorporating prior knowledge regarding relative spatial relationships and geometric features, the proposed method reduces the processing time while ensuring the accuracy of component segmentation and center coordinates calculation.
4. A method of component recognition and segmentation is proposed to be applicable throughout the approach phase. The difficulty of component recognition increases as the distance decreases since the target spacecraft may become partially visible in the field of view. A component recognition method that considers anomalies caused by shadow occlusion and control deviations is proposed to accurately and reliably match each detected region with a corresponding component (main body, each solar panel, etc.), utilizing a rotated rectangular fitting coefficient and the relative spatial relationship between the regions.

The rest of this paper is organized as follows: Section 2 introduces the preliminaries. The details of the proposed method are described in Section 3. In Section 4, the performance and computational efficiency of the proposed method are evaluated and analyzed. Finally, conclusions are drawn in Section 5.

## 2. Preliminaries

During the approach phase, the LOS angles from the servicing spacecraft to the target spacecraft are calculated through the camera pinhole model and perspective projection. The LOS angles assist the relative navigation system in achieving autonomous relative navigation. In this section, the fundamentals of the reference coordinate systems and the phases of rendezvous are introduced.

### 2.1. Reference Coordinate Systems

#### 2.1.1. The Image Coordinate System and Pixel Coordinate System

**Figure 1:**
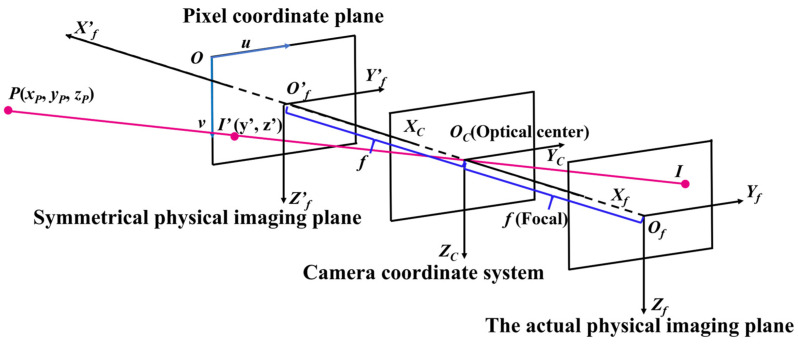
Conversion from the pixel coordinate system to the camera coordinate system.

A pixel coordinate plane *O*–*uv* is defined as follows. As shown in Figure 1, its origin *O* is located in the top left corner of the image, and the *Ou* and *Ov* are the horizontal axis and vertical axis of the image, respectively. A pixel point is represented as **x** = [*u*, *v*]^Τ^ in the pixel coordinate plane. The origin of the image coordinate system is located at the principal point, which is the intersection of the optical axis and the detector plane. The direction outward from the symmetrical physical imaging plane is the positive direction of the *O*′*_f_X*′*_f_* axis, and the *O*′*_f_Y*′*_f_* axis and *O*′*_f_Z*′*_f_* axis are along the horizontal and vertical of the pixel coordinates, respectively.

#### 2.1.2. The Camera Coordinate System

The camera coordinate system *O_C_*–*X_C_Y_C_Z_C_* is fixed to the main body of the servicing spacecraft. As shown in Figure 1, the origin of the camera coordinate system is located at the optical center of the camera. The axis *O_C_X_C_* is along the direction of the optical axis, and the *O_C_Y_C_* and *O_C_Z_C_* axes are parallel to the *Ou* and *Ov* axes of the pixel coordinate system, respectively. The projection of the point *O_C_* onto the actual physical imaging plane is the principal point *O_f_*, which is located at the center of the actual physical imaging plane. The axes *O_f_X_f_* and *O_f_Y_f_* are parallel to *O_C_X_C_* and *O_C_Y_C_*, respectively. The distance between *O_C_* and *O_f_* represents the focal length of the camera, denoted as *f*.

### 2.2. Coordinate Conversion

**Figure 2:**
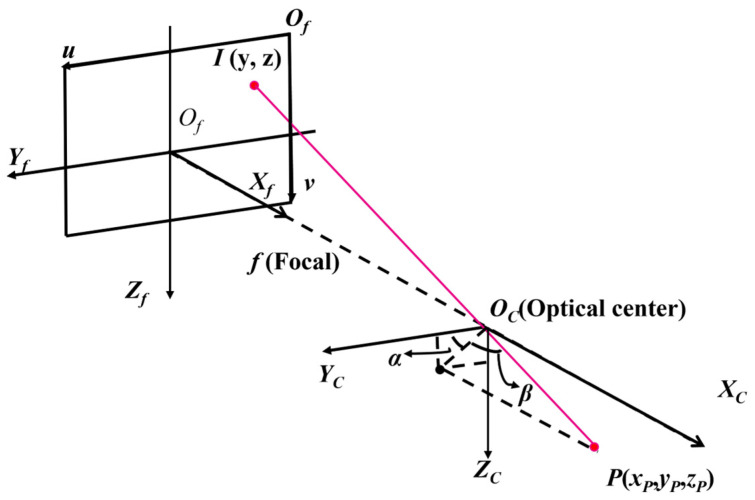
Definition of the LOS angles in the camera coordinate system.

As illustrated in Figure 1, the target point is denoted as *P* and its coordinates in the camera coordinate system are $\left(x_{P},y_{P},z_{P}\right)$. Its perspective projection on the image plane is represented by point *I′*, with coordinates (*y′*, *z′*) in the image coordinate system. The coordinates of the target in the pixel coordinate system are denoted as (*u_c_*, *v_c_*). These are transformed to the image coordinate system through the following relationship:

(1) $$\left\{\begin{array}{c} y^{\prime }=dy\times (u_{c}-u_{0})\\ z^{\prime }=dz\times (v_{c}-v_{0}) \end{array}\right.$$

where (*u*_0_, *v*_0_) are the coordinates of the image center in pixels, and *dy*, *dz* represent the physical dimensions of a pixel in the horizontal and vertical directions, respectively. According to the perspective projection model, the relationship between the image coordinates and the camera coordinates is given by:

(2) $$\left\{\begin{array}{c} y_{P}=x_{P}\frac{y^{\prime }}{f}\\ z_{P}=x_{P}\frac{z^{\prime }}{f} \end{array}\right.$$

where *f* is the focal length of the camera. As shown in Figure 2, the LOS vector from the camera optical center *O_C_* to the target point *P* can be expressed in the camera coordinate system as $v_{c}={\left[x_{P},y_{P},z_{P}\right]}^{Τ}$. Using Equation (2), the same directional vector can be equivalently represented in the normalized image plane (where $x_{P}=1$) as $v_{n}={\left[1,\frac{x^{\prime }}{f},\frac{z^{\prime }}{f}\right]}^{Τ}$. This vector is normalized to obtain the unit LOS vector:

(3) $$i_{P}=\frac{v_{n}}{\left\|v_{n}\right\|}$$

where $i_{P}$ points from the camera towards the target in the camera coordinate system. In this study, the LOS orientation is defined by two angles in the camera coordinate system: the out-of-plane angle *β* and the in-plane angle *α*, as illustrated in Figure 2. The out-of-plane angle *β* is defined as the angle between the unit vector $i_{P}$ and the image plane *O_f_*–*X_f_Y_f_Z_f_*. The in-plane angle α is defined as the angle between the projection of the unit vector $i_{P}$ onto the image plane and the *O_C_Y_C_* axes. They can be calculated as:

(4) $$\left\{\begin{array}{c} \beta =\sin^{-1}(i_{P,x})\\ \alpha =\tan^{-1}(\frac{i_{P,z}}{i_{P,y}}) \end{array}\right.$$

where $i_{P,x}$, $i_{P,y}$, and $i_{P,z}$ are the components of the unit vector $i_{P}$ along the *O_C_X_C_*, *O_C_Y_C_*, and *O_C_Z_C_* axes, respectively.

### 2.3. Overview of the Rendezvous Phases

**Figure 3:**
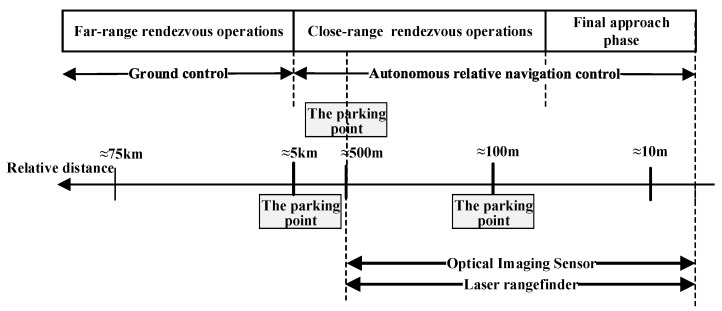
Phases of rendezvous operations: from far-range to final approach.

Figure 3 illustrates the phases of rendezvous operations, from far range to final approach. The far-range rendezvous phase is conducted by ground control using orbit prediction, during which the spacecraft performs a series of preplanned orbital maneuvers. When the distance to the target decreases to around 5 km, the servicing spacecraft initiates the close-range rendezvous. From this point, it initiates autonomous relative navigation and navigates to the target at a distance of several kilometers by utilizing real-time measurements of LOS information. This work focuses on the terminal segment of this phase, starting at a relative distance of 500 m. Using real-time measurements of the range and LOS information, the servicing spacecraft approaches the target spacecraft in a straight line from the front until it reaches 10 m, at which point the final docking sequence is typically initiated.

## 3. Proposed Method

**Figure 4:**
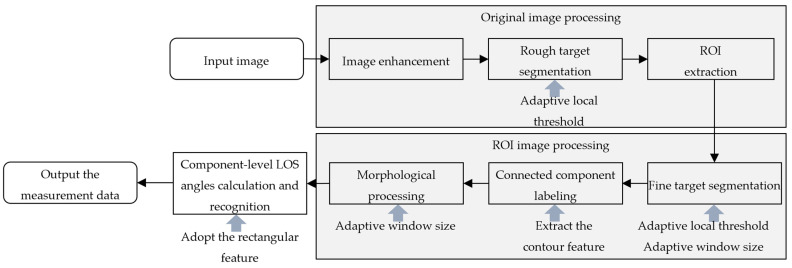
The main idea of the proposed component-level LOS angles measurement method.

To address the challenges mentioned in the introduction, a continuous component-level LOS angle measurement method is proposed. To tackle the challenge of varying target sizes, the proposed method follows a coarse-to-fine processing logic. Specifically, a rough target segmentation is achieved, and the ROI is first extracted from the original image. Subsequently, a fine target segmentation based on adaptive thresholding, connected component labeling and morphological processing is performed on the ROI. In order to adapt to the dynamic changes in the target size, the parameters of these operations are seamlessly and adaptively changed according to the ROI size. To enhance the robustness to illumination changes, the preprocessing technique is applied to the image to enhance the salient features of the target. Adopting the features that are insensitive to lighting changes to replace texture features can reduce the influence of lighting. The proposed method calculates the center coordinates using contour features and recognizes the component based on the rectangular geometric feature. Finally, the efforts made by the method to achieve real-time processing with high frame rates are adjusting the processing complexity according to distance and hardware acceleration for image preprocessing and convolution operations using the Graphics Processing Unit (GPU) of NVIDIA Jetson TX2. This significantly reduces the computing load and increases the processing speed. The main idea of the proposed method is illustrated in Figure 4.

### 3.1. Image Enhancement

The lighting conditions in space are complex and variable: the overall brightness of the target changes with distance, and due to the unidirectional sunlight, the surface of the target often shows a distinct brightness gradient. The low contrast of spacecraft materials makes the challenge more pronounced. Excessive lighting conditions or prolonged camera exposure times can improve the contrast. However, they may result in the loss of crucial target structure information. Although cameras usually have an automatic exposure function, their performance in complex space lighting conditions still has limitations. Therefore, achieving reliable feature extraction within a limited range of lighting intensity requires a method for lighting-robust contrast enhancement. In this work, the adaptive gamma correction with weighting distribution (AGCWD) [26] method is used. It dynamically combines the global statistical characteristics of the image with the gray values of local pixels, thereby enhancing the details of dark regions more naturally and intelligently while avoiding over-enhancement and brightness saturation. In addition, to reduce the blurring and artifacts caused by the amplification of high-frequency noise in target features, Gaussian filtering is performed prior to the feature enhancement process.

The AGCWD algorithm is applied to the denoised image to enhance contrast. When contrast is adjusted through gamma correction with fixed parameters, different images will show the same intensity change. This cannot be adapted to different scenarios. To address this issue, the probability density function (PDF) of each gray level in a digital image is calculated as:

(5) $$p_{l}=\frac{n_{l}}{\left(M\times N\right)}$$

where *n_l_* denotes the pixel number of the gray level *l*. *M* × *N* is the total number of pixels in the image, and the length *N* and width *M* of the image are given in terms of the number of pixels, respectively. Moreover, inspired by the Recursively Separated and Weighted Histogram Equalization (RSWHE) method [27], a weighting distribution function is used to subtly adjust the statistical histogram, which is defined as:

(6) $$w_{l}=p_{\max}{\left(\frac{p_{l}-p_{\min}}{p_{\max}-p_{\min}}\right)}^{\alpha }$$

where α is the adjusted parameter, and *p_max_* and *p_min_* are the maximum and the minimum probability density of the statistical histogram, respectively. The cumulative distribution function (CDF) is calculated as:

(7) $$cdf_{l}=\frac{\sum_{k=0}^{k=l}w_{k}}{\sum_{k=0}^{k=l_{\max}}w_{k}},0\leq k\leq l_{\max}$$

**Figure 5:**
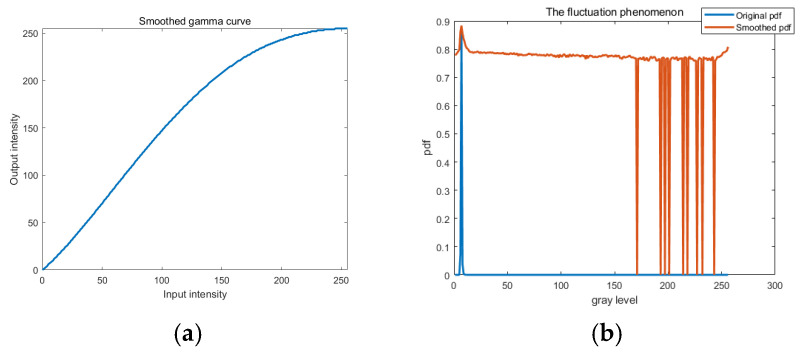
(**a**) Smoothed gamma curve and (**b**) the fluctuation phenomenon.

After generating the *cdf_l_* of the input image, the adaptive gamma correction is derived by:

(8) $$l_{out}=l_{\max}{\left(\frac{l}{l_{\max}}\right)}^{\gamma }=l_{\max}{\left(\frac{l}{l_{\max}}\right)}^{1-cdf_{l}}$$

where *l_max_* is the maximum gray level of the input gray image. The gray level *l* of each pixel in the input image is transformed as *l_out_* of each pixel in the enhanced image according to Equation (8). The enhanced image *I_E_* is generated after applying adaptive gamma correction with a weighting distribution. For dimmed input images, the gray values of most pixels are densely concentrated at a relatively low level. According to (8), *γ* is less than 1, which expands the regions of the image with lower gray levels and compresses the regions with higher gray levels. As shown in Figure 5a, lower gray levels are expanded slightly, while medium and higher gray levels are compressed significantly. By applying the weighting distribution function, the fluctuation phenomenon can be smoothed out as shown in Figure 5b, thus mitigating the over-enhancement caused by gamma correction. In Figure 5b, the original image exhibits obvious peaks in its PDF at low gray levels and large gaps at high gray levels. Gamma correction broadens these peaks and partially fills the gaps. However, due to sparse high-brightness regions with many unused levels, noticeable depressions appear in the PDF after correction. This indicates that the original gray-level distribution is highly sparse at high intensities, and the enhancement compresses low-probability levels and fills gaps, resulting in valleys in the smoothed distribution.

**Figure 6:**
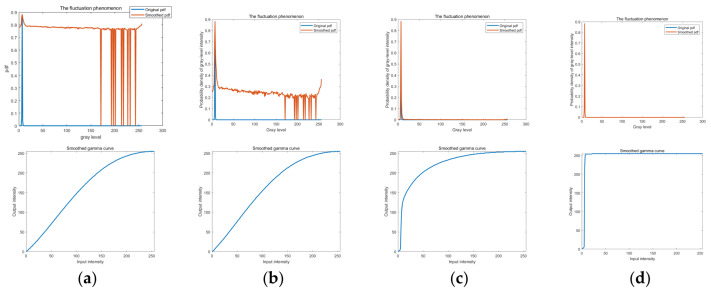
Smoothed gamma curves and fluctuation phenomena with different *α* values. (**a**) *α* = 0.01; (**b**) *α* = 0.1; (**c**) *α* = 0.5; (**d**) *α* = 1.

The fluctuation phenomenon and the smoothed gamma curve of different α values are displayed in Figure 6. When α is 0.01, the low and medium gray levels are expanded, which means that the details of the target are maintained. When α is 0.5, the low gray level is compressed significantly, which causes severe distortion. Therefore, α is set as 0.01 based on our experiments. The performance of the enhancement verifies that this technique is suitable for enhancing details of space images under different brightness conditions.

### 3.2. ROI Extraction

#### 3.2.1. Image Segmentation by Adaptive Local Thresholding

After generating the enhanced image *I_E_*, a thresholding technology is employed to achieve binarization. This step aims to extract the complete target from the background. Global thresholding methods, which are effective under uniform lighting, often perform poorly in real-world conditions with varied illumination. To overcome this limitation, a local adaptive threshold method is proposed. The process of binarization is represented as:

(9) $$I^{BW}=\left\{\begin{array}{c} 1,I_{E}(x,y)>T(x,y)\\ 0,I_{E}(x,y)<T(x,y)\; \end{array}\right.$$

where *T*(*x*, *y*) is the threshold value of the pixel at the coordinates (*x*, *y*) in the threshold image *T*, *I_E_* (*x*, *y*) is the gray value of the pixel at the coordinates (*x*, *y*) in the enhanced image *I_E_* and *I^BW^* is the binarized image. The initial local adaptive threshold based on sensitivity is calculated as follows.

(10) $$T_{init}(x,y)=s\times \frac{1}{m_{b}\times n_{b}}\times \sum_{x=1}^{m_{b}}\sum_{y=1}^{n_{b}}I_{E}(x,y)$$

where *T_init_*(*x*, *y*) denotes the initial threshold value of the pixel at the coordinates (*x*, *y*). *s* is the scale factor based on sensitivity, and *m_b_*, *n_b_* are the dimensions of the moving window. Experimental results indicate that selecting a large window size helps to retain as much target information as possible. Therefore, *m_b_* and *n_b_* are set to one-fourth of the number of rows and columns in the input image, respectively. The sensitivity is a scalar in the range [0, 1] that indicates sensitivity towards thresholding more pixels as the foreground. A high sensitivity value leads to thresholding more pixels as the foreground, at the risk of including some background pixels. Information entropy quantifies the amount of information within an image based on information theory. A higher information entropy value indicates that there is more information present in the image. By utilizing information entropy as sensitivity, the segmentation threshold can be adaptively determined based on the information content within the image. Based on a bright-foreground prior, sensitivity *H* ϵ [0, 1] is mapped to a scaling factor *s* ϵ [0.6, 1.6] through Equation (11). Lower *H* increases the threshold to prevent the bright background from being mistakenly segmented as foreground. Higher *H* decreases it to retain weak foregrounds. The constant 0.6 ensures *H* = 0.5 yields *s* = 1.1, providing a symmetric adjustment ±0.5 range around this default for flexible adjustment without excessive under- or over-segmentation.

(11) s=0.6+(1−H)

(12) $$H=-\sum_{l=0}^{l_{\max}}p_{l}\log_{2}(p_{l})$$

where *p_l_* represents the probability intensity of the gray level *l* among the total pixel number *M* × *N*. Finally, the initial threshold is standardized within the data range of [0, *l_max_*].

(13) $$T(x,y)=\min(T_{init}(x,y),l_{\max})$$

where *l_max_* is the maximum gray level of the input gray image.

#### 3.2.2. Region of Interest (ROI) Extraction

To ensure high-precision component center localization and component segmentation, the ROI that encloses the target is extracted. After thresholding, connected component labeling is performed using the seed filling method [28]. In medium-range and close-range imaging, the target is much larger than the stars in space, and each star in this image occupies very few pixels. To eliminate interference from stars, the proposed approach sets the area threshold to 0.1% of the entire image size. If the area of a connected region is smaller than this threshold, the pixel values within the region are set to 0. Since there may be multiple regions with components, the external rectangle that encloses all the regions is selected as the ROI. Compared with the original image, it takes less time to perform component center localization and component recognition on the ROI with fewer pixels.

### 3.3. Region Detection

Local adaptive thresholding is a key prerequisite for achieving precise component recognition and segmentation. As the distance decreases, the number of pixels occupied by the target increases gradually. The fixed size of the convolution kernel is only applicable within a certain range of distances. To precisely extract the geometric features of the target, the thresholding is optimized by adjusting the kernel size parameter. The size of the ROI is multiplied by a specific coefficient to serve as the convolution kernel size. It means that the window size changes in accordance with the size of the ROI. The rectangular kernel size (*m_th_*, *n_th_*) of the threshold is set as:

(14) $$\begin{array}{l} m_{th}=W_{ROI}\times 0.4\\ n_{th}=H_{ROI}\times 0.4 \end{array}$$

where the height *H_ROI_* and width *W_ROI_* of the ROI are given in terms of the number of pixels.

After thresholding, all the connected components are labeled by seed filling. Due to interference from material properties and non-uniform illumination conditions, the target cannot be completely segmented. The closing operation is used to facilitate the connections within the components. The dimensions of the local window *m_c_* and *n_c_* in the closing operation directly affect the strength of these connections. Small window sizes cannot facilitate connections within the components effectively. However, a large window size may also strengthen the connections between components. To strengthen intra-component connections while minimizing inter-component connections, the processing window size is normalized to the height of the ROI as the dimensional reference. In addition, the smaller the window size is, the faster they process. Therefore, in consideration of the trade-off between performance and processing speed, the window size (*m_c_*, *n_c_*) of the closing operation is set as:

(15) $$\begin{array}{l} m_{c}=H_{ROI}\times 0.01\\ n_{c}=H_{ROI}\times 0.01 \end{array}$$

In practical applications, the target is partially captured in the image where the servicing spacecraft approaches the target spacecraft very closely. At far-range, control deviation or shadow occlusion of the servicing spacecraft can also cause partial visibility of the target spacecraft in images. In this paper, the ratio of the overall width to height of the target is used to identify these cases. After performing thresholding, connected component labeling and the closing operation, the measured width $W_{Tar}^{M}$ and height $H_{Tar}^{M}$ of the target spacecraft are obtained. The details of the recognition method will be introduced in Section 3.6.

Subsequently, the opening operation is used to separate each component. Sometimes, a small window size cannot break the strong connections between components. Conversely, an excessively large window size may lead to degradation of the target region. To eliminate inter-component connections, the window size (*m_o_*, *n_o_*) of the opening operation is set experimentally as:

(16) $$\begin{array}{l} m_{o}=W_{ROI}\times 0.01\\ n_{o}=W_{ROI}\times 0.01 \end{array}$$

After morphological processing, a binarized image the size of *W_ROI_* × *H_ROI_* is generated, containing the separated component regions.

### 3.4. Calculation of the LOS Angle to the Component

The center coordinates (*u_c_*, *v_c_*) of a region are calculated based on the first- and zero-order raw moments, as follows:

(17) $$M_{00}=\sum_{x}\sum_{y}I(x,y)$$

(18) $$M_{10}=\sum_{x}\sum_{y}xI(x,y)$$

(19) $$M_{01}=\sum_{x}\sum_{y}yI(x,y)$$

(20) $$u_{c}=\frac{M_{10}}{M_{00}},v_{c}=\frac{M_{01}}{M_{00}}$$

where *I*(*x*, *y*) represents the gray value at the coordinate (*x*, *y*) in the region, *M*_00_ is the zero-order raw moment of the region, and *M*_01_ and *M*_10_ are the first-order raw moments of the region.

The center coordinates (*u_c_*, *v_c_*) of each component are calculated in the ROI, which should be restored in the original image. The target center coordinates ($u_{c}^{\prime }$, $v_{c}^{\prime }$) in the original image are calculated using Equation (21):

(21) $$\begin{array}{l} u_{c}^{\prime }=u_{c}+u_{topleft}\\ v_{c}^{\prime }=v_{c}+v_{topleft} \end{array}$$

where the coordinates (*u_topleft_*, *v_topleft_*) represent the top left point of the ROI in the original image. Next, the LOS angles are generated through coordinate conversion introduced in Section 2.2.

### 3.5. Calculation of the Rectangular Fitting Coefficient to the Component

After generating the center coordinates for all regions, we still cannot recognize which component each region belongs to. To solve this, a component recognition method is necessary to determine the specific component each region represents. Solar panels have a distinct rectangular shape, whereas the main body is irregular. Therefore, the rectangularity of a region contour can be used to distinguish between them. In this study, the discrepancy between a detected region and its best-fit rectangle is measured to quantify its rectangularity. In this paper, the minimum bounding rectangle is not used to fit the region because a rotation angle in the original region can introduce bias, resulting in an inaccurate rectangularity measurement. To address the limitations, Rosin [29] adopts the image ellipse. The orientation of the rectangle is derived by fitting an ellipse to match the first- and second-order central moments of the region. The sides of the rectangle are then defined by the lengths and directions of the major and minor axes of this ellipse. The lengths of the rectangle’s sides *a* and *b* are calculated as:

(22) $$a=\sqrt{\frac{6[\mu_{20}+\mu_{02}+\sqrt{{\left(\mu_{20}-\mu_{02}\right)}^{2}+4\mu_{11}^{2}}]}{\mu_{00}}}$$

(23) $$b=\sqrt{\frac{6[\mu_{20}+\mu_{02}-\sqrt{{\left(\mu_{20}-\mu_{02}\right)}^{2}+4\mu_{11}^{2}}]}{\mu_{00}}}$$

where *μ_pq_* is the (*p* + *q*)th-order central moment of the region. The data is translated by ($u_{c}^{\prime }$, $v_{c}^{\prime }$) and rotated by −*θ* to make the descriptor invariant to rotation and translation. The center coordinates can be calculated as in Equation (21), and the orientation *θ* of the data is derived as:

(24) $$\theta =\frac{1}{2}\tan^{-1}\frac{2\mu_{11}}{\mu_{20}-\mu_{02}}$$

The rectangularity is evaluated by calculating the rectangular fitting coefficient *C_rf_*.

(25) $$C_{rf}=1-\frac{\left(A_{1}+A_{2})+(A_{3}-A_{2}\right)}{A_{3}}=1-\frac{A_{1}+A_{3}-2A_{2}}{A_{3}}$$

where *A*_1_ is the area of the detected region, *A*_3_ is the area of the fitted rectangle and *A*_2_ is the area of the detected region where the fitted rectangle overlaps. The discrepancy between the region and the fitted rectangle consists of two parts: the area of the region outside the rectangle *A*_1_ − *A*_2_, and the area of the rectangle that is not filled by the region *A*_3_ − *A*_2_. The discrepancy is normalized by the area of the fitted rectangle and then subtracted from 1 to generate the fitting coefficient.

**Figure 7:**
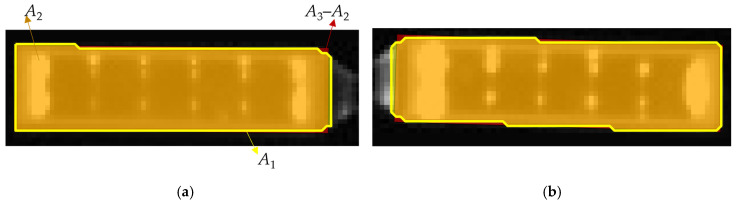
Rectangular fitting results of solar panels: (**a**) left solar panel; (**b**) right solar panel. *A*_1_ is the area of the detected region, *A*_3_ is the area of the fitted rectangle and *A*_2_ is the area of the detected region where the fitted rectangle overlaps.

As shown in Figure 7, due to edge artifacts, the yellow contour of the detected region is irregular after thresholding and morphological operations. Compared with the fitted rectangle highlighted in green, the discontinuous lines are observed along the edges of the original region. The rectangular fitting coefficient calculated through Equation (25) measures the discrepancies between the fitted rectangle and the detected region.

### 3.6. Component Recognition

**Figure 8:**
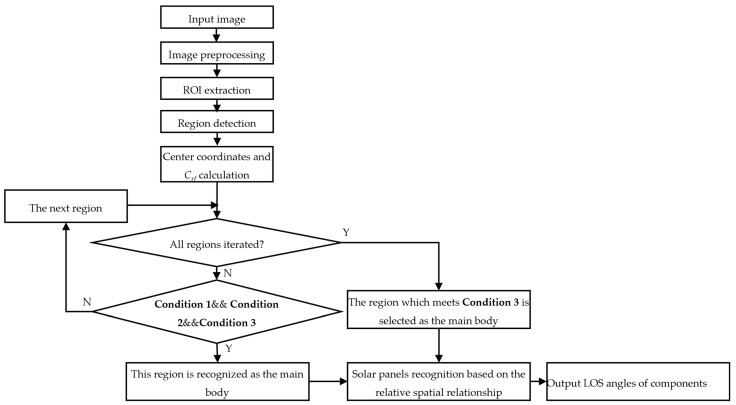
Flow chart of the proposed component recognition method based on center coordinates and rectangle fitting coefficients (*C_rf_*).

A component recognition method based on geometric features and component spatial relationships is proposed. As illustrated in Figure 8, the main body is recognized first, and the solar panels are recognized according to their relative spatial relationship to the main body. The algorithm iterates through all detected regions to recognize the main body. The selection conditions are as follows:

(1) Condition 1: The absolute difference between each *C_rf_* value and the minimum value among all *C_rf_* values is to be below 0.5. This threshold is used to distinguish regions of low rectangularity, including the main body, from rectangular solar panels.
(2) Condition 2: The area of this region should exceed 10% of the area of the largest region. Detected regions are sorted by area, with the solar panel being the largest, followed by the main body. This area threshold excludes smaller regions.
(3) Condition 3: The horizontal center coordinate of the region must be closest to the mean horizontal center coordinates of all regions. Using the mean horizontal center, this condition selects the region that is closest to the target in the horizontal direction.

Three conditions are summarized as follows:

(26) $$\left\{\begin{array}{c} |C_{rf,i}-\min(C_{rf,1},C_{rf,2},\ldots ,C_{rf,N_{R}})|<0.5\\ A_{i}>\max(A_{1},A_{2},\ldots ,A_{N_{R}})\times 0.1\\ u_{c,i}^{\prime }-\mathrm{mean}(u_{c,1}^{\prime },u_{c,2}^{\prime },\ldots ,u_{c,N_{R}}^{\prime })=\min(|u_{c,i}^{\prime }-\mathrm{mean}(u_{c,1}^{\prime },u_{c,2}^{\prime },\ldots ,u_{c,N_{R}}^{\prime })|) \end{array}\right.\;,\;\;\;\;\;\;i\in \{1,\;2,\;3,\ldots ,\;N_{R}\}$$

where *i* is the region index, and *N_R_* is the total number of detected regions. *C_rf_*_,*i*_ and *A_i_* are the rectangular fitting coefficient and the area of the *i*-th region, respectively. $u_{c,i}^{\prime }$ represents the horizontal center coordinate of the *i*-th region.

Consistent and reliable spatial configurations of components are critical for recognizing the main body when primary features are ambiguous. Therefore, if no region satisfies all three conditions simultaneously, the algorithm selects the region that fulfills Condition 3.

After recognizing the main body, other regions are divided into two groups on the left and right sides based on the center of the main body. For each side, the region that is closest to the center of the main body in the horizontal direction is determined as the solar panel on that side.

After recognizing the center coordinates of three components, the final center coordinates of the main body are refined to eliminate interference from the antenna. Based on the prior information that two solar panels are symmetrically arranged along the vertical center line of the main body, the vertical center coordinate of the main body lies on the perpendicular bisector of the line connecting the centers of the two solar panels. The final horizontal center coordinate of the main body is the same as the horizontal coordinate initially calculated by Equations (20) and (21).

We assume that the servicing spacecraft tracks the center of the main body from 500 m to 150 m, and tracks the center of another component from 150 m to 10 m. A parameter *M_T_* is introduced to denote tracking modes, where *M_T_* = 0, 1, 2 represent tracking the left solar panel, the main body and the right solar panel, respectively. During approach, the target is usually centered, and detected regions are typically three or more. However, unexpected obstructions caused by orbit control deviations or shadow occlusion, or ultra-close-range approach, may reduce the count to fewer than three. Based on the number of detected regions, the recognition method is classified into four cases: no valid region, a single region, two regions, and three or more regions.

(1) If *N_R_* = 0, which means that there are no regions being detected, the processing goes back to the first step, waiting for the next image.
(2) If *N_R_* = 1, it indicates that one region is being detected. There are three situations that could cause this.
  Situation 1: Due to the unidirectional light source, some occluded components located in the shadow are invisible in the image.
  Situation 2: The servicing spacecraft may experience control deviation during the approach phase, causing the target spacecraft to deviate from the expected relative navigation window. Consequently, the optical image sensor can only capture part of it.
  Situation 3: The servicing spacecraft is very close to the target spacecraft, and there is only one tracking target component in the image. The tracking target component is centered in the image. For the three situations considered, the key characteristics observed in the data are summarized in Table 1. √ indicates that the characteristic listed in the row is present in the situation corresponding to that column. × indicates that the characteristic is not present.
  In this work, the servicing spacecraft approaches the target spacecraft from the front and the target does not have a significant attitude change during the approach phase. The width-to-height ratio of the target in the image changes if the target is partially visible. Assuming that the ground truth for the target satellite size and geometry is known, the true width-to-height ratio can be calculated. To exploit this characteristic, we detect whether the ratio of the measured target width ($W_{Tar}^{M}$) to the measured target height ($H_{Tar}^{M}$) is close to the known true ratio. The ratio error criterion is calculated as follows:
  (27) $$\frac{\left|\frac{W_{Tar}^{M}}{H_{Tar}^{M}}-\frac{W_{Tar}^{T}}{H_{Tar}^{T}}\right|}{\frac{W_{Tar}^{T}}{H_{Tar}^{T}}}<10\%$$
  where $W_{Tar}^{T}$ and $H_{Tar}^{T}$ are the true target width and height, respectively. The second characteristic is identified by detecting the presence of continuous non-zero pixels along the edges of the image.
  A situation can be classified as shadow occlusion if the first characteristic is present but the second is absent. In this situation, the method cannot recognize which component it is. Consequently, the LOS angle of the whole region is referred to as that of the target. If the first two characteristics persist for fifty consecutive frames, the servicing spacecraft is considered to be very close to the target, and this state is locked. This means that the next input frame will enter the ultra-close-range approach directly. The detected region can be referred to as the tracking component according to *M_T_*, and the LOS angle of the tracking component is output. Otherwise, it indicates that the servicing spacecraft is experiencing control deviation, and the LOS angle of the region is output as that of the target.
(3) If *N_R_* = 2, it indicates that two regions are being detected. Similar to the case where *N_R_* = 1, this may result from situations of shadow occlusion, control deviation, or an ultra-close-range approach. The first step is to determine which abnormal situation the servicing spacecraft is in. For the situations of shadow occlusion and control deviation, two regions are merged into one, and the central LOS angle of the merged region is output as the central LOS angle of the target spacecraft. At ultra-close range, the tracking target switches from the main body to another component, which remains centered. As distance decreases, both components appear in the image. The region that is located in the center of the image is referred to as the tracking component, and the other component is recognized based on the relative spatial relationship to the tracking component in the horizontal direction. And then, output the LOS angles of the other component and the tracking target component according to *M_T_*.
(4) If *N_R_* ≥ 3, it means that three or more regions are detected. The proposed component recognition method is applied to recognize each component. It outputs the LOS angles of the main body, the left solar panel and the right solar panel.

**Figure 9:**
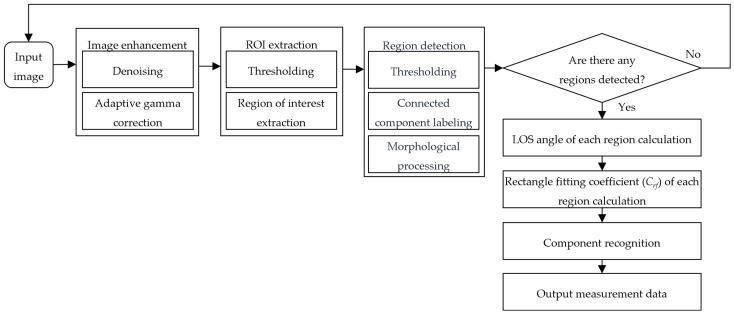
Main steps of the proposed method.

Finally, the main steps of the proposed method are displayed in Figure 9.

### 3.7. Adaptability Discussion for Other Targets

The proposed method performs a layer-by-layer analysis using traditional image processing techniques. Its core procedure begins with image preprocessing for noise reduction and enhancement, followed by adaptive thresholding and region detection to extract the ROI. Within the ROI, a series of finer operations, including thresholding, region detection, and morphological operations, is then applied to recognize key components. Importantly, these procedures are driven by geometric relationships extracted from the image itself, rather than by any assumption of structural symmetry. This fundamental characteristic enables the method to be applicable to other spacecraft designs. However, since the component recognition method is based on the relative spatial configuration between components, adjustments must be made at this point for asymmetric targets featuring just a single solar panel.

Based on the assumption that the servicing spacecraft approaches the target spacecraft from the front, the proposed method is also applicable to targets with rectangular Synthetic Aperture Radar (SAR) antennas and asymmetric targets. A target equipped with rectangular SAR antennas refers to one where the solar arrays are symmetrically arranged relative to the target’s vertical centerline, and its rectangular SAR antennas are oriented toward the Earth to transmit and receive radar signals for Earth observation imaging. An asymmetric target refers to a type of target that has a solar panel on only one side. The adaptability of the proposed method to these two types of targets is described as follows.

The main components of asymmetric targets are a main body and a single solar panel on one side of the main body. Firstly, the observed images are taken as input, and the image preprocessing operations, including denoising and contrast enhancement, are conducted. Subsequently, thresholding is carried out to segment the target from the background. According to the threshold of the area, the small regions are removed and the external rectangle that encloses all detected regions is selected as the ROI. The area threshold is set to 0.1% of the entire image area, which is the same as the original setting. Next, more precise thresholding, connected component labeling and morphological operations are performed on the ROI. The conditions used to recognize the main body of the asymmetric target are different from those of a symmetric target. The center of the main body is not on the perpendicular bisector of the target. Therefore, the conditions are represented as:

(28) $$\left\{\begin{array}{c} |C_{rf,i}-\min(C_{rf,1},C_{rf,2},\ldots ,C_{rf,N_{R}})|<0.5\\ A_{i}>\max(A_{1},A_{2},\ldots ,A_{N_{R}})\times 0.1 \end{array}\right.,\;\;\;\;\;\;\;i\in \{1,\;2,\;3,\ldots ,\;n\}$$

If no region satisfies all the above conditions simultaneously, the region meeting the second condition is selected as the main body. Next, the remaining region is recognized as the solar panel.

For targets with SAR antennas, the main task is to eliminate the influence of SAR antennas on component segmentation. Similarly, performing image preprocessing operations and adaptive thresholding to obtain the ROI. The SAR antennas are installed on the bottom of the main body, and the area they occupy when observed from the front is extremely small. When the servicing spacecraft approaches the target from the front, only the side profile of the rectangular SAR antenna can be observed. Therefore, they can be easily eliminated during morphological opening operations, which do not affect the critical components’ segmentation. The LOS angle calculation and component recognition methods are consistent with those for symmetrical targets.

## 4. Performance Demonstration

### 4.1. Experiment Design

Autonomous relative navigation requires that the method provide the LOS angle of each component accurately and continuously. Specifically, the method must support real-time processing, adaptability to spatial illumination changes, and high-precision persistent measurements. In this section, the performance of the proposed method is evaluated through comprehensive testing across three distinct datasets. Firstly, images with realistic scene lighting and space backgrounds are simulated by an image simulator software. These images are used to test the capability of achieving high-precision and persistent measurement during the approach phase. Secondly, a darkroom simulation platform that accounts for realistic spatial lighting is built to generate actual images and validate the robustness of the method under varying distances, brightness levels, and attitudes. Thirdly, the method is deployed on the NVIDIA Jetson TX2, and its real-time performance is evaluated through ground-based semi-physical simulation. The computational complexity and processing speed of the proposed method using simulated images and actual images are also quantitatively assessed.

**Figure 10:**
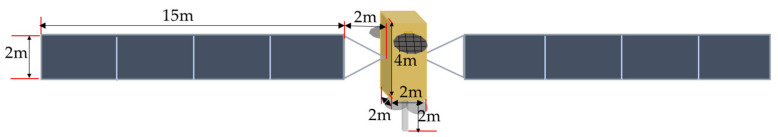
Diagram of the first type of spacecraft.

**Figure 11:**
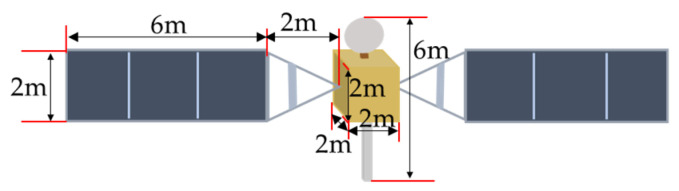
Diagram of the second type of spacecraft.

Considering the most common structural features of satellites, two types of satellite models are used to simulate the imaging process of space targets. The first type of spacecraft consists of a main body, two deployable solar panels laterally attached to the main body, two antennas mounted on the front and rear sides of the main body, and two antennas mounted on the bottom of the main body. The model of this spacecraft is made to perform a dark room experiment, and the size ratio of the model to the actual spacecraft size is 70:1. The second type of spacecraft consists of a main body, two deployable solar panels laterally attached to the main body, and an antenna mounted on the top of the main body. The actual dimensions of two spacecraft are displayed in Figure 10 and Figure 11.

The database consists of three datasets. The first dataset of the two types of spacecraft is generated using the image simulator. A ground semi-physical system is constructed to simulate the entire approach phase. The image simulator receives attitude and orbit data, as well as orientation information of the sun and moon. It first determines the relative position of the target through coordinate conversion, then computes the gray values of the target pixels based on the sun position vector and the diffuse reflection. To replicate degraded imaging conditions, the simulation incorporates both Gaussian and salt-and-pepper noise models, ultimately rendering the target spacecraft in its spatial environment. During this phase, a total of 2110 simulated images from 500 m to 10 m are generated in real time. The details of ground semi-physical simulation are introduced in Section 4.5.

The second dataset is generated by sampling from the first dataset. An image of the target spacecraft is sampled at 1 m intervals in actual distance. This dataset consists of 495 simulated images. The validity study of this persistent component-level LOS angles measurement with distance variations is conducted on the second dataset. The details about the effectiveness verification of the proposed method are introduced in Section 4.3.

**Figure 12:**
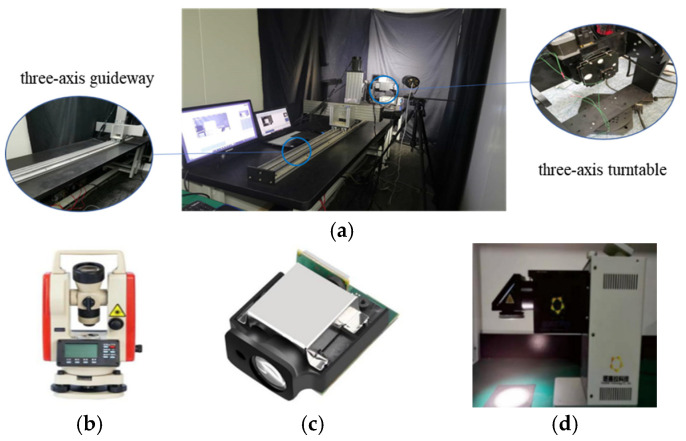
Dark room experimental instruments: (**a**) three-axis guideway and three-axis turntable; (**b**) theodolite; (**c**) laser rangefinder; (**d**) solar simulator.

**Figure 13:**
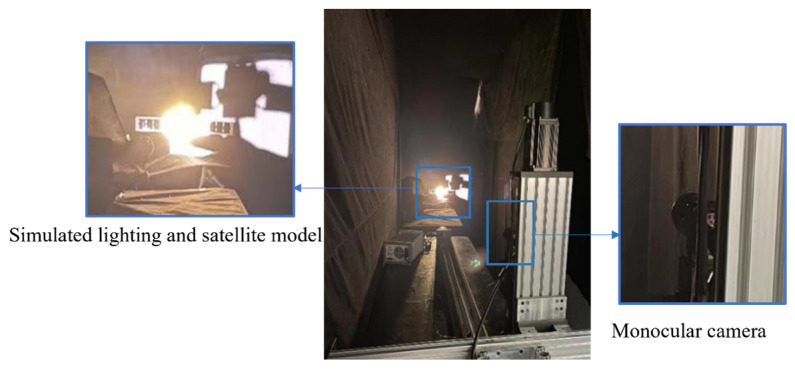
Experimental setup in the dark room.

The third dataset is generated through an experimental platform, which is constructed to capture actual images for the first type of spacecraft. A target model is produced by replicating every component of the actual spacecraft, the solar panels and the main body surface in a 70:1 proportion, using the same materials as the actual spacecraft. The experimental instruments are shown in Figure 12, and their measurement ranges and accuracy are summarized in Table 2. The platform is equipped with a three-axis guideway and a three-axis turntable. A Gigabit Ethernet camera, fitted with a fixed 8 mm lens, is mounted at the end of the three-axis guideway and connected to a computer. It provides a 79° × 79° field of view, with a sensor that captures images at a native resolution of 2048 × 2048 pixels and outputs 12-bit grayscale data. A laser rangefinder (Jiaqi, Ningbo, China) and a theodolite (Boif, Beijing, China) measure distance and angle, respectively. A solar simulator is employed to simulate the solar illumination. To evaluate the robustness of the method, various scenarios are simulated in the dark room to capture images, including different distances, exposure times, aperture sizes, and relative attitude angles. The image acquisition process in the dark room is described as follows. As shown in Figure 13, a black background is used to simulate a dark, deep-space environment. In order to simulate the actual solar illumination and direction in space, the solar simulator is positioned from the side towards the target model. The distance variation from 70 m to 10 m is simulated by moving the three-axis guideway. To obtain images of different brightness, a fixed distance of 2.85 m (corresponding to an actual 199.50 m) is set. Firstly, the aperture is set to f/1.4, and one image is collected at each exposure time. Then this process was repeated for the apertures of f/2.0 and f/2.8, respectively. In order to obtain images under different relative attitudes, a three-axis turntable is adopted to simulate the pitch, yaw and roll angle changes in the target model relative to the line of sight of the camera. The specific ranges of the distance, aperture, exposure time and relative attitude angle are shown in Table 3. Systematic validation across the entire range of parameters in Table 3 confirms that the method is not highly dependent on specific settings and maintains stable performance, demonstrating its reliability and practicality.

### 4.2. Evaluation Metrics

The measurement accuracy of LOS angles is evaluated by computing the error between the measured and ground-truth LOS vectors. For the simulated images, the ground-truth center coordinates of the components in the observed image are known. For the images generated in the dark room, the ground-truth center coordinates of the components are not directly available but can be extracted in the observed image. Subsequently, both the measured and ground-truth LOS vectors are calculated as Equation (3) based on the coordinate conversion introduced in Section 2.2. Finally, the line-of-sight vector error (LOSVE) is computed as:

(29) $$LOSVE=\arccos<i_{p}^{M},i_{p}^{G}>=\arccos(\frac{i_{p}^{M}\cdot i_{p}^{G}}{\left|i_{p}^{M}\right|\left|i_{p}^{G}\right|})$$

where $i_{p}^{M},i_{p}^{G}$ represent the measured and ground-truth LOS vectors, respectively.

To evaluate segmentation performance, Pixel Accuracy (PA) and Intersection over Union (IoU) are calculated by comparing the segmentation results of the proposed method with the manually annotated ground-truth labels. Both metrics are calculated using TP (True Positive), FP (False Positive), TN (True Negative), and FN (False Negative) from the confusion matrix. In the context of component segmentation, *TP* of each component represents the area of the target component in the ground-truth label that is correctly segmented as the same target component in the segmentation result; *FP* of each component denotes the area of the non-target components in the ground-truth label that are falsely segmented as the target component in the segmentation result; *FN* denotes the area of the target component in the ground-truth label that is falsely segmented as the non-target components in the segmentation result; and *TN* represents the area of the non-target components in the ground-truth label that are correctly segmented as the same non-target components in the segmentation result. *PA* measures the percentage of correctly predicted pixels for a specific target component. Specifically*, PA_i_* is the proportion of the number of correctly predicted pixels to the total number of pixels for the *i*-th class. Mean Pixel Accuracy (MPA) is calculated by averaging the *PA* values across all four classes, indexed as *k* = 0 (background), *k* = 1 (left solar panel), *k* = 2 (right solar panel), and *k* = 3 (main body). It comprehensively presents the overall segmentation accuracy of the proposed method. According to the reference [30], the *PA* values of different classes and *MPA* are calculated as:

(30) $$PA_{i}=\frac{TP_{i}+TN_{i}}{TP_{i}+TN_{i}+FP_{i}+FN_{i}}$$

(31) $$MPA=\frac{1}{k+1}\sum_{i=0}^{k}PA_{i}$$

*IoU_i_* is the ratio of the intersection area of the segmentation results and the ground-truth labels to the union area of the segmentation results and the ground-truth labels for the *i*-th class. Mean Intersection over Union (MIoU) is calculated by averaging *IoU* values across all classes. It reflects the average overlap between the segmentation results and the ground-truth labels of the proposed method. The *IoU* values of different classes and *MIoU* can be calculated as:

(32) $$IoU_{i}=\frac{TP_{i}}{TP_{i}+FP_{i}+FN_{i}}$$

(33) $$MIoU=\frac{1}{k+1}\sum_{i=0}^{k}IoU_{i}$$

### 4.3. Effectiveness Verification of the Proposed Method

**Figure 14:**
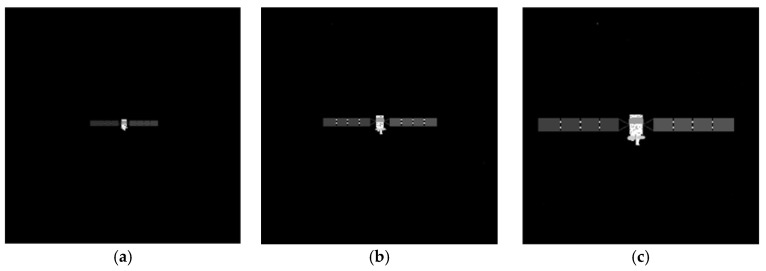

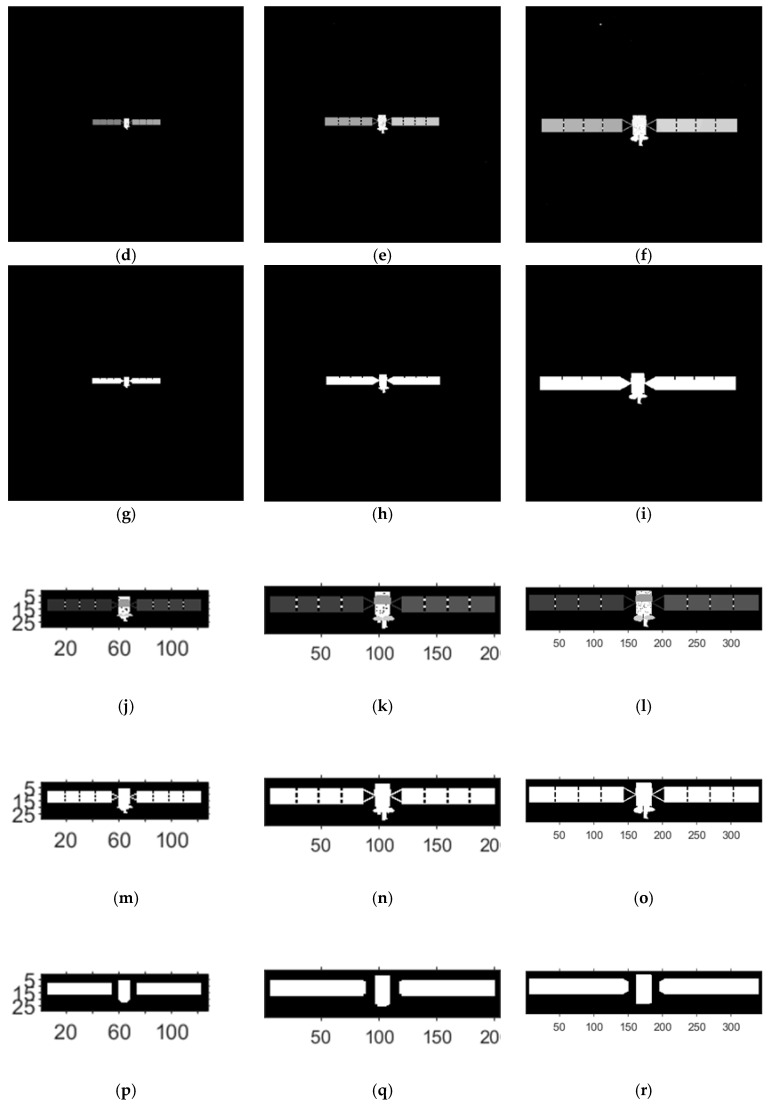
Evolution of simulated images at 500 m, 300 m, and 100 m through the proposed adaptive processing pipeline, including adaptive gamma correction, adaptive thresholding, ROI extraction, ROI thresholding, and ROI morphological operations. (**a**) Original image at 500 m; (**b**) original image at 300 m; (**c**) original image at 100 m; (**d**) enhanced image at 500 m; (**e**) enhanced image at 300 m; (**f**) enhanced image at 100 m; (**g**) binarized image at 500 m; (**h**) binarized image at 300 m; (**i**) binarized image at 100 m; (**j**) extracted ROI image at 500 m; (**k**) extracted ROI image at 300 m; (**l**) extracted ROI image at 100 m; (**m**) binarized ROI image at 500 m; (**n**) binarized ROI image at 300 m; (**o**) binarized ROI image at 100 m; (**p**) the ROI image at 500 m after morphological operations; (**q**) the ROI image at 300 m after morphological operations; (**r**) the ROI image at 100 m after morphological operations.

**Figure 15:**
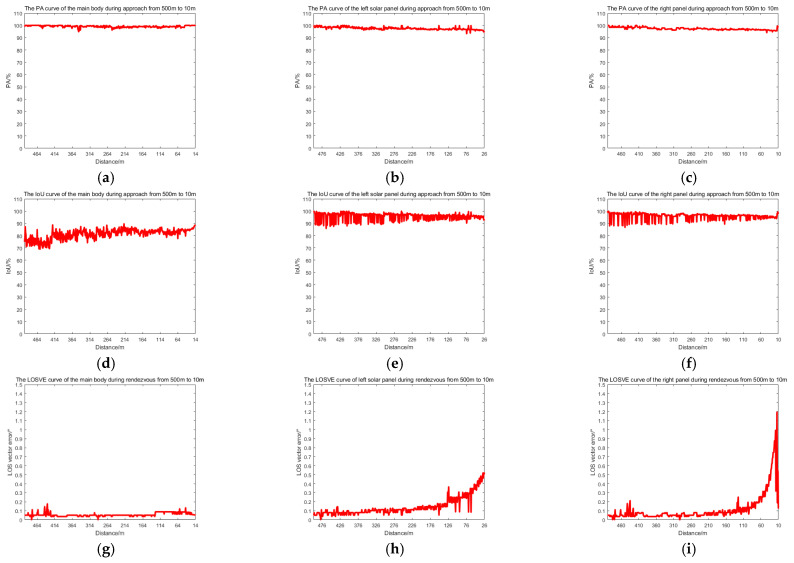
*PA*, *IoU* and *LOSVE* curves of each component during approach from 500 m to 10 m. (**a**) *PA* curve of the main body during approach from 500 m to 10 m; (**b**) *PA* curve of the left solar panel during approach from 500 m to 10 m; (**c**) *PA* curve of the right solar panel during approach from 500 m to 10 m; (**d**) *IoU* curve of the main body during approach from 500 m to 10 m; (**e**) *IoU* curve of the left solar panel during approach from 500 m to 10 m; (**f**) *IoU* curve of the right solar panel during approach from 500 m to 10 m; (**g**) *LOSVE* curve of the main body during approach from 500 m to 10 m; (**h**) *LOSVE* curve of left solar panel during approach from 500 m to 10 m; (**i**) *LOSVE* curve of the right solar panel during approach from 500 m to 10 m.

**Figure 16:**
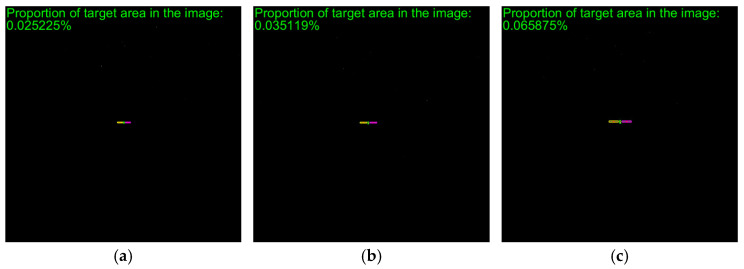

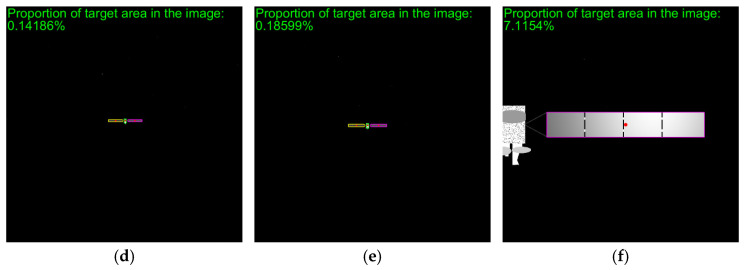
Visualization of center coordinates and component segmentation in simulated images at varying distances: (**a**) 500 m; (**b**) 400 m; (**c**) 300 m; (**d**) 200 m; (**e**) 100 m; (**f**) 10 m.

**Figure 17:**
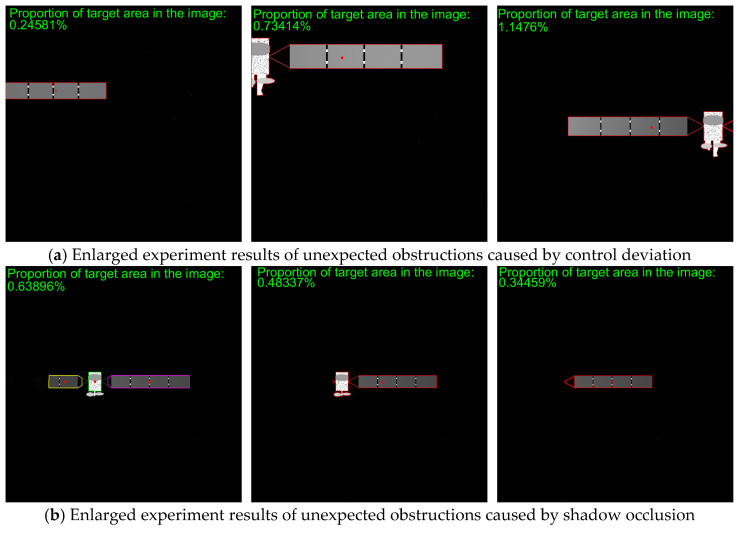
Visualization of center coordinates and component segmentation in simulated unexpected obstructions: (**a**) control deviation; (**b**) shadow occlusion.

To verify the effectiveness of the proposed method, simulated images from 500 m to 10 m are processed. The proposed method includes adaptive thresholding, adaptive gamma correction and morphological operations to mitigate resolution-dependent performance loss. The simulated images at distances of 500 m, 300 m, and 100 m are selected, and their results from the image processing steps, including adaptive gamma correction and adaptive thresholding, are enlarged and presented in Figure 14d–i. For different distances, the images are effectively enhanced by adaptive gamma correction, while targets are completely segmented from the background by adaptive thresholding. The binarized images in Figure 14j–r are then processed through ROI extraction, ROI thresholding, and ROI morphological operations, and the results are presented in Figure 14j–r. For different target scales resulting from decreasing distance, adaptive thresholding precisely segments the targets, and morphological operations with seamless parameter adaptation effectively separate the components. The *PA*, *IoU* and *LOSVE* curves for each component during the approach phase are displayed in Figure 15. *PA* and *IoU* measure the segmentation accuracy of each class. Their stability indicates that the algorithm maintains consistent component segmentation capability across distances. The *LOSVE* values of the main body remain consistently low from 500 m to 10 m, not exceeding 0.2°. The *LOSVE* values of the left solar panel are not greater than 0.6°. As the distance decreases and the tracking target component is replaced with the right panel, the *LOSVE* values of the right panel increase. In some individual frames, the *LOSVE* values exceed 1°. This is because the horizontal center coordinate of the right solar panel is influenced by the bracket used to connect the main body and the solar panels. However, this has little influence on the navigation system when persistent and real-time data updates are ensured. The segmentation results at distances of 500 m, 400 m, 300 m, 200 m, 100 m and 10 m are presented in Figure 16. The red “*” indicates the centers of the components. The proportion of the target area in the image is shown in the top-left corner of the image. It is obvious that the target spacecraft occupies more pixels as the distance decreases. According to Figure 15 and Figure 16, the proposed method can achieve the continuous measurement of component-level LOS angles from 500 m to 10 m. In addition, this work considers two unexpected obstructions caused by control deviation and shadow occlusion, as described in Section 3.6. Figure 17 shows the experimental results, which demonstrate that the proposed method can identify these obstructions and output the LOS angles of the target center or its component centers.

As shown in Table 4, the second to fourth columns present the average *PA* values of all testing images from 500 m to 10 m for each component, while the fifth column presents the *MPA* values of all classes. The *PA* values of the solar panels are beyond 97.36%. Overall, the *MPA* value of the proposed method can reach 98.51%. The sixth to eighth columns show the *IoU* values and the ninth column shows the *MIoU*. The *IoU* of the solar panels is beyond 95.72%, and the *MIoU* of the proposed method is 93.25%. According to columns 10–12 of Table 4, the mean line of sight vector error (MLOSVE) values is less than 0.2°. Experimental results confirm the capability of the method in high-precision and persistent measurement of the component-level LOS angles during the dynamic approach.

**Figure 18:**
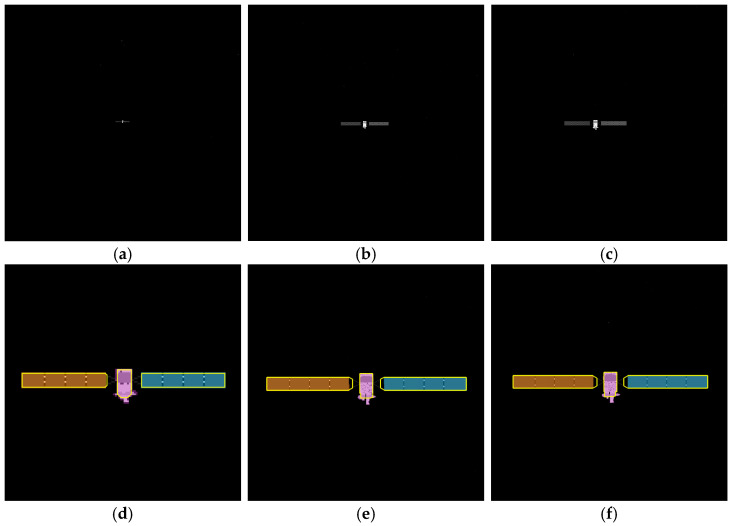

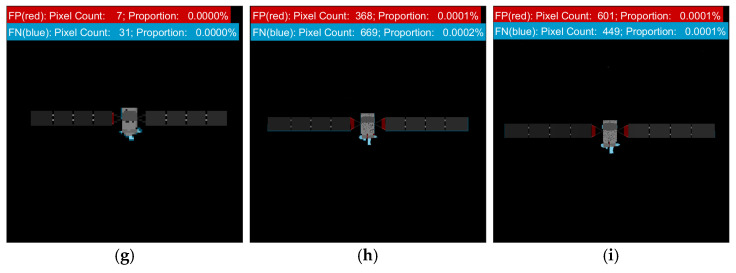
Visualization and analysis of false positive and false negative errors in representative segmentation cases. (**a**) Original image at 497 m; (**b**) original image at 141 m; (**c**) original image at 62 m; (**d**) ground truth and prediction contours overlay at 497 m; (**e**) ground truth and prediction contours overlay at 141 m; (**f**) ground truth and prediction contours overlay at 62 m; (**g**) segmentation error overlay at 497 m; (**h**) segmentation error overlay at 141 m; (**i**) segmentation error overlay at 62 m.

To analyze the workings of the method and identify critical steps, three representative cases are selected for analysis: a well-segmented example, an image with a high number of foreground FP, and an image with a high number of foreground FN. As shown in Figure 18, we present three visualizations for each case: the original image in the first row, the original image overlaid with ground truth and prediction contours in the second row, and the original image with highlighted FP (red) and FN (blue) regions in the third row. The corresponding pixel counts and proportions of FP and FN are also provided in the figure.

As illustrated, FP pixels are mainly concentrated around the connecting bracket between the solar panels and the main body, as this structure is not fully eliminated by the morphological erosion operation. FN pixels, on the other hand, primarily appear at the antenna located beneath the main body and along the edges of the target. The former arises from the erosion process removing the protruding part below the body, while the latter is caused by the erosion operation eroding the target boundaries.

This highlights the trade-off between noise suppression and boundary preservation in erosion. Further improvements could involve tuning morphological parameters or integrating boundary-aware refinement.

### 4.4. Robustness Evaluation in Terms of Distance, Brightness and Attitude Variation

**Figure 19:**
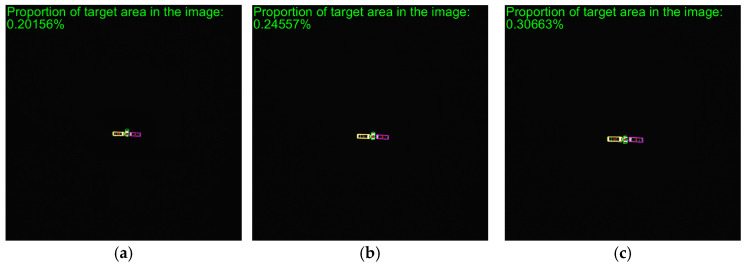

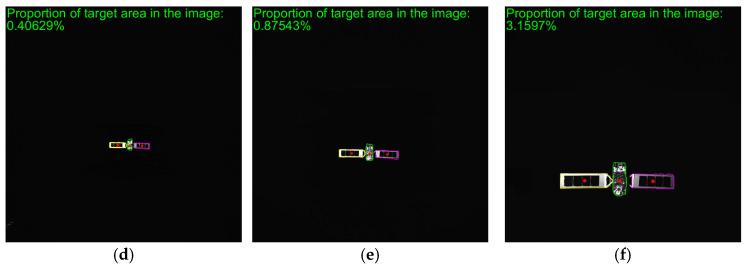
Visualization results of center coordinates and component segmentation are presented in actual images at different distances: (**a**) 70 m; (**b**) 62 m; (**c**) 55 m; (**d**) 32 m; (**e**) 22 m; (**f**) 12 m.

As shown in the experimental setup in Figure 13, the close-range approach is simulated by moving the camera along a calibrated three-axis guideway. The actual images are captured as the camera moves from 1 m to 0.1 m. This simulates the process where the servicing spacecraft approaches the target spacecraft from a distance of 70 m to 10 m. The *MPA* and *MIoU* are 88.10% and 86.46%, respectively. The *MLOSVE* values for the left solar panel, right solar panel and the main body are 0.1263°, 0.1252°, and 0.1548°, respectively. Specific statistical results are presented in Table 5, and the visualization results of center coordinates and component recognition at different distances of 70 m, 62 m, 55 m, 32 m, 22 m, and 12 m are shown in Figure 19. In conclusion, by using actual images, the proposed method enables the accurate and persistent measurement of the component-level LOS angles during the approach.

**Figure 20:**
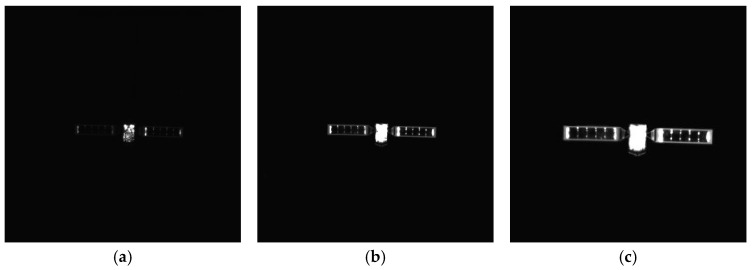

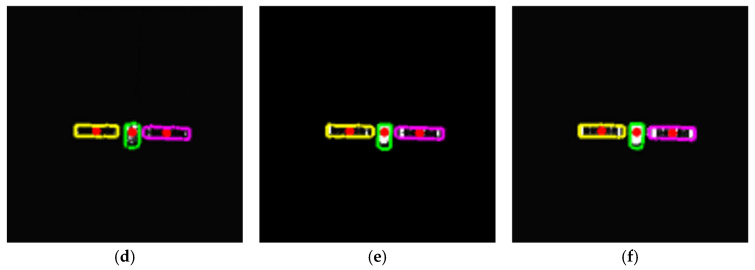
Experimental results at 2.85 m and f/2.8 with various exposure times: (**a**) original image at 4.7 ms; (**b**) original image at 34.5 ms; (**c**) original image at 69 ms; (**d**) corresponding segmentation result for (**a**); (**e**) corresponding segmentation result for (**b**); (**f**) corresponding segmentation result for (**c**).

The variation in brightness is simulated mainly by changing the aperture size and exposure time. A large aperture and a long exposure time result in high brightness. On the other hand, using a small aperture and a short exposure time decreases the image brightness. The test results at a relative distance of 2.85 m (i.e., an actual distance of 199.50 m), with an aperture of f/2.8 and exposure times of 4.7 ms, 34.5 ms and 69 ms, are selected and shown in Figure 20. To ensure clear visibility, the original images are enlarged and shown in Figure 20a–c. To adapt to brightness changes, our method adjusts the image brightness through adaptive gamma correction. Based on the visualization results in Figure 20d–f, from very low to high illumination conditions, the proposed method can achieve the measurement of component-level LOS angles. At a distance of 2.85 m, the *MLOSVE*, *PA* and *IoU* values for apertures of f/1.4, f/2 and f/2.8, as well as for exposure times from 4.7 ms to 69 ms are presented in Table 6. Taking the first row as an example, it displays the average values of *PA*, *IoU* and *LOSVE* for an aperture of f/1.4 at a distance of 2.85 m. The third to fifth columns display the average *PA* values calculated across all exposure times for the left solar panel, right solar panel and main body, respectively. The sixth column provides the average of all classes. The *MPA* values for three aperture sizes are 89.24%, 89.01% and 88.39%, respectively. Due to the significant rectangular shape of solar panels, the *IoU* is high and stable. The *MIoU* values for three aperture sizes are 88.82%, 88.57% and 88.04%, respectively. As shown in the eleventh to thirteenth columns, the *MLOSVE* values of the proposed method are all below 0.2°. Overall, it can be concluded that this method adapts to different lighting conditions to ensure effective measurement of component-level LOS angles.

In this work, the servicing spacecraft approaches the target spacecraft from the front, and the target maintains a relatively stable attitude without significant variations during the approach phase.

**Figure 21:**
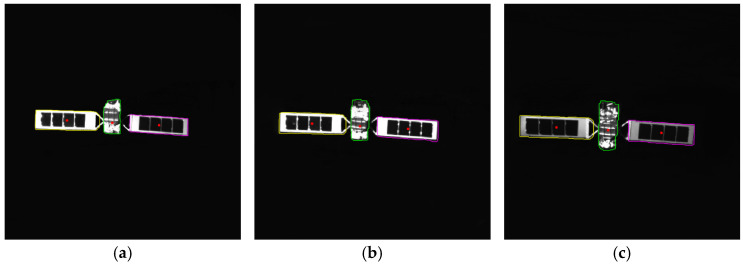

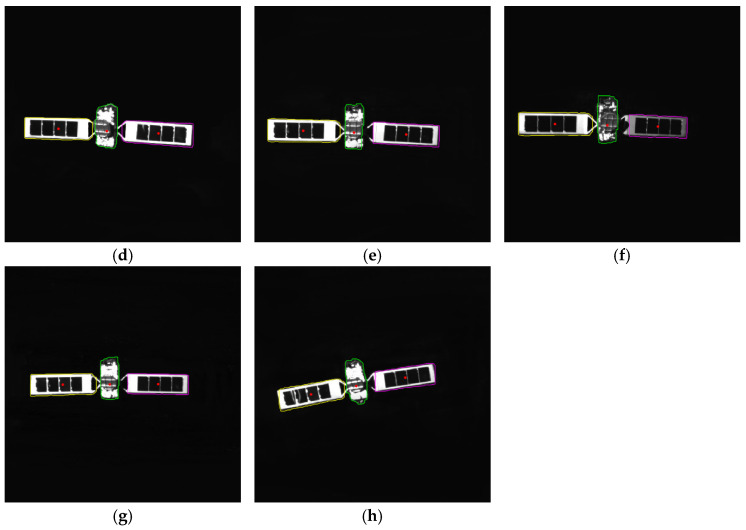
Experimental results at 25 m: (**a**) pitch angle −10°; (**b**) pitch angle 0°; (**c**) pitch angle 10°; (**d**) yaw angle −10°; (**e**) yaw angle 0°; (**f**) yaw angle 10°; (**g**) roll angle 0°; (**h**) roll angle 10°.

To validate the impact of attitude changes on the method, a three-axis turntable is used to simulate scenarios where the target is rotated by 10° in pitch, yaw, and roll angles relative to the line of sight of the camera. Each rotational degree of freedom was considered independently in separate experiments. Specifically, the relative pitch angle varied from 0° to both +10° and −10°. The relative yaw angle is changed from 0° to both +10° and −10°, while the roll angle is rotated to +10°. As shown in Figure 21, the method successfully achieves accurate component segmentation and center localization under these attitude variations.

### 4.5. Navigation System and Ground Semi-Physical Simulation

The proposed method is deployed on the NVIDIA Jetson TX2 for subsequent performance evaluation experiments. In this section, the real-time performance of our method is evaluated through a ground semi-physical simulation.

**Figure 22:**
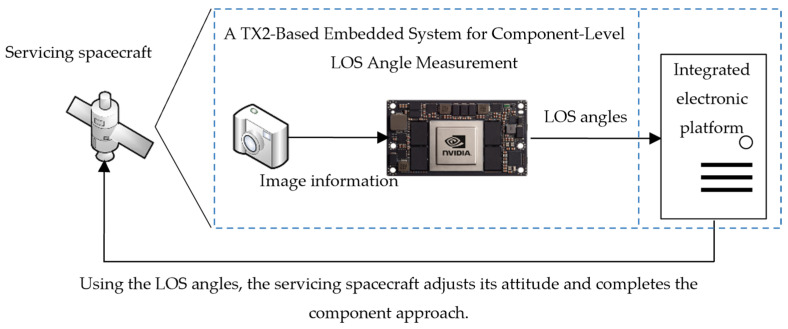
Architecture diagram of TX2 embedded system.

Firstly, an architecture diagram of the TX2 embedded system in a practical scenario is presented in Figure 22. In the navigation system, the hardware system in the servicing spacecraft contains three parts: a visual information acquisition module, a visual information processing module and a navigation and control integrated electronic platform. The core of the visual information processing module contains the proposed component-level LOS angles measurement method. On-orbit information processing not only demands real-time and high-precision algorithms but also imposes stringent constraints on power consumption, size, and computational power. The NVIDIA Jetson TX2 high-performance embedded platform not only excels in computational capabilities and energy efficiency but also offers robust peripheral interface compatibility and comprehensive software support. In conclusion, the Jetson TX2 serves as a viable hardware platform for implementing our method in information processing and real-time decision-making. The proposed method has been successfully deployed and functionally validated on this platform.

Subsequently, the crucial step is to establish communication between each module. In this system, a network port camera is used to capture the images. UDP is used as the communication protocol. The GNC module is one of the components of the integrated electronic platform. The measured LOS angles from TX2 assist the integrated electronic platform in adjusting the attitude of the servicing spacecraft and performing the component approach. The GNC module commonly uses FPGA architecture, so it often communicates with external devices through serial ports. Although the TX2 does not have a direct serial interface, it provides multiple Universal Asynchronous Transmission (UART) pins, which can be configured as serial ports through algorithms. The transmitted data consists primarily of the LOS angles and several operational status indicators, which are represented as bit-level signals. As a result, the RS-422 protocol with high anti-interference is selected as the standard for data transmission between TX2 and the integrated electronic platform. In summary, the system composition shown in Figure 22 can be described as follows:

(1) TX2 is connected to a camera to receive images in real time.
(2) The TX2 processes received images to derive the LOS angles.
(3) The measurement data is transmitted from TX2 to the integrated electronic platform. It aims to assist the attitude and orbit control system in completing the component approach to the target spacecraft.

**Figure 23:**
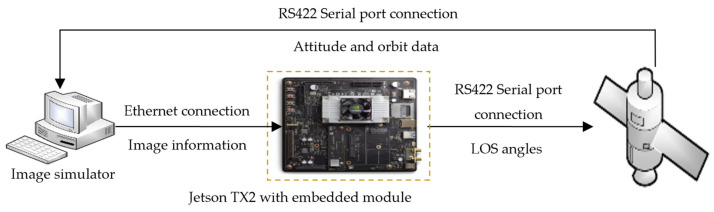
Schematic of the ground semi-physical simulation system.

Secondly, to verify the real-time performance and reliability of measuring LOS angles and data transmission, the ground semi-physical simulation system is established and shown in Figure 23. It consists of an image simulator, the NVIDIA Jetson TX2 and the integrated electronic platform. Specifically, the image simulator simulates the images of the target spacecraft on a Personal Computer (PC). The TX2 processes images using embedded software to achieve real-time acquisition of LOS angles. As a navigation control simulator, the integrated electronic platform is responsible for simulating trajectories in Satellite Tool Kit (STK) 5.0 based on the received measurement information. The image simulator receives attitude and orbit data from the integrated electronic platform, as well as orientation information of the sun and moon. It produces the space target images. In this way, a closed-loop is formed.

According to the analysis of system requirements, the steps of constructing the ground semi-physical simulation platform are concluded as follows:

(1) The PC running the image simulator is connected to the TX2 network port through a network cable, enabling established UDP communication between the two devices. The image simulator transmits image data to the TX2 at a rate of four frames per second.
(2) The serial port module of the integrated electronic platform is connected to the USB port of the TX2, establishing RS-422 protocol communication between the two serial ports. TX2 outputs the measurement information to the integrated electronic platform.
(3) The serial port module of the integrated electronic platform is connected to the PC through a serial-to-USB cable.

**Figure 24:**
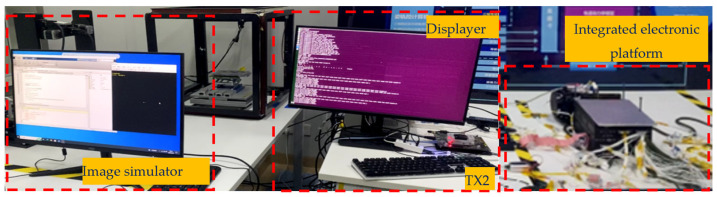
Hardware components of the ground semi-physical simulation system.

Finally, the actual experimental platform is shown in Figure 24.

Using the measured results from the TX2, the integrated electronic platform simulates the position and attitude of the servicing spacecraft through STK 5.0. Subsequently, the component approach is accomplished based on the dynamic simulator along with the attitude and orbit control algorithm. According to experiments, the TX2 embedded system can receive image data from the network port at a 4 Hz rate and process the images at a 3 Hz rate. The ground semi-physical simulation experiment verifies that the TX2 with an embedded system has good real-time performance, reliable data transmission, and accurate measurement of the component-level LOS angles.

### 4.6. Computational Complexity and Processing Speed

The proposed method has the advantages of low computation complexity and a high processing frequency. It runs on a PC with an i7-7800X Central Processing Unit (CPU) and 16 GB Random-Access Memory (RAM). It achieves average processing frequencies of 1.7 Hz with simulated images and 2 Hz with captured images. We implemented it on an NVIDIA Jetson TX2 embedded platform, achieving a real-time update rate of 3 Hz. Resource measurements show that CPU usage across all six cores stays below 50% utilization, and memory usage is 46%, since most operations except for adaptive gamma correction are performed pixel-wise, and the denoising, thresholding and morphological operations can be accelerated through the GPU. These results confirm that the method is lightweight and energy efficient. The main challenge for space applications is radiation resistance. Due to its simple algorithm and low resource needs, the method can be easily ported to other embedded processors, including radiation-hardened models used in space, with only minor changes. This issue can also be mitigated through software-based error correction, redundancy in system design, and automatic retransmission of data.

## 5. Conclusions

This paper presents a method for on-orbit measurement of the component-level LOS angles with seamless parameter adaptation. After image denoising and enhancement, regions of each component are detected through connected component labeling. The components are recognized based on the geometric features and relative spatial relationships. The effectiveness of the method is verified using the simulated images; the *MPA* and *MIoU* of the proposed method are 98.51% and 93.25%, respectively. The robustness of this method in terms of distance, brightness and attitude variation is verified using the actual images. The *MPA* and *MIoU* values of the method using actual images are greater than 88.10% and 86.46%, respectively. The *MLOSVE* is less than 0.2° for the simulated images and less than 0.2° for the actual images captured in the dark room. Moreover, the proposed method exhibits low computational complexity, with pixel-wise and convolution operations that are highly parallelizable to improve GPU acceleration. On the TX2 platform, the method is capable of processing data at 3 Hz. For future on-orbit missions, it is crucial to protect electronic systems from space radiation and Single Event Upset (SEU). Our future work will prioritize software-level robustness to complement hardware design. This will include error detection and correction methods, along with redundancy on both the software and system architecture levels to prevent data corruption and transmission failures. Another key improvement will be a feedback mechanism that allows the software to automatically send error reports or request data retransmission from the onboard system when problems are found.

## Figures and Tables

**Table 1:** The characteristics of the observed data in three situations.

		Shadow Occlusion	Control Deviation	Ultra-Close-Range Approach
Characteristic 1	Partial Visibility of the Target	√	√	√
Characteristic 2	Target at Image Edge	×	√	√
Characteristic 3	Prolonged Continuous Occurrence	×	×	√

**Table 2:** Experimental instruments and parameters.

Instruments	Parameters
Three-axis guideway	Resolution: 0.05 m X-axis length ranging from 0 to 6 m Y-axis length ranging from 0 to 0.7 m Z-axis length ranging from 0 to 0.5 m
Three-axis turntable	Accuracy: 0.0005° Rotating 360° along the X, Y and Z axes
Laser rangefinder	Accuracy: ±1 mm Ranging from 0.05 to 80 m
Solar simulator	Spectral ranging from 320 to 800 nm Spot size is 50 mm

**Table 3:** Experimental scene setting.

Scenes	Range
Relative distance	70 m~10 m
Aperture	f/1.4, f/2.0, f/2.8
Exposure time	4.7 ms, 6.9 ms, 13.8 ms, 20.7 ms, 27.6 ms, 34.5 ms, 41.4 ms, 48.3 ms, 55.2 ms, 62.1 ms, 69 ms
Pitch	−10°~10°
Yaw	−10°~10°
Roll	0°~10°

**Table 4:** Performance evaluation in *MLOSVE*, *PA*, and *IoU* of the proposed method from 500 m to 10 m.

Relative Distance	PA (%)			MPA (%)	IoU (%)			MioU (%)	MLOSVE (°)		
	Left Solar Panel	Right Solar Panel	Main Body		Left Solar Panel	Right Solar Panel	Main Body		Left Solar Panel	Right Solar Panel	Main Body
500 m~10 m	97.59	97.36	99.10	98.51	95.72	95.96	81.36	93.25	0.1433	0.1143	0.0562

**Table 5:** Performance evaluation on *MLOSVE*, *PA*, and *IoU* of the proposed method from 70 m to 10 m.

Relative Distance	*PA* (%)			*MPA* (%)	*IoU* (%)			*MIoU*(%)	*MLOSVE* (°)		
	Left Solar Panel	Right Solar Panel	Main Body		Left Solar Panel	Right Solar Panel	Main Body		Left Solar Panel	Right Solar Panel	Main Body
70 m~10 m	82.02	87.49	82.92	88.10	82.01	87.36	76.55	86.46	0.1263	0.1252	0.1548

**Table 6:** *PA, IoU* and *MLOSVE* of the proposed method at various aperture sizes and exposure times at a distance of 2.85 m.

Aperture	Exposure Time	*PA* (%)			*MPA* (%)	*IoU* (%)			*MioU* (%)	*MLOSVE* (°)		
		Left Solar Panel	Right Solar Panel	Main Body		Left Solar Panel	Right Solar Panel	Main Body		Left Solar Panel	Right Solar Panel	Main Body
f/1.4	4.7~69 ms	86.73	84.74	85.49	89.24	86.43	84.36	84.52	88.82	0.0675	0.0351	0.0706
f/2.0	4.7~69 ms	89.04	86.90	80.10	89.01	88.48	86.55	79.28	88.57	0.0515	0.0322	0.0764
f/2.8	4.7~69 ms	87.97	87.42	78.18	88.39	87.86	86.95	77.37	88.04	0.0595	0.0326	0.0710

## Data Availability

The original contributions presented in the study are included in the article, and further inquiries can be directed to the corresponding authors.

## Abbreviations

The following abbreviations are used in this manuscript:

LOS	Line of Sight
LiDAR	Light Detection and Ranging
CCD	Charge - Coupled Device
GNSS	Global Navigation Satellite Systems
3D	Three-Dimensional
CNNs	Convolutional Neural Networks
YOLO	You Only Look Once
GNC	Guidance, Navigation and Control
DOF	Degrees of Freedom
ROI	Region of Interest
LVLH	Local Vertical Local Horizontal
AGCWD	Adaptive Gamma Correction with Weighting Distribution
PDF	Probability Density Function
RSWHE	Recursively Separated and Weighted Histogram Equalization
CDF	Cumulative Distribution Function
SAR	Synthetic Aperture Radar
LOSVE	Line-of-Sight Vector Error
PA	Pixel Accuracy
IoU	Intersection over Union
TP	True Positive
FP	False Positive
TN	True Negative
FN	False Negative
MPA	Mean Pixel Accuracy
MIoU	Mean Intersection over Union
MLOSVE	Mean Line-of-Sight Vector Error
UART	Universal Asynchronous Transmission
PC	Personal Computer
STK	Satellite Tool Kit
CPU	Central Processing Unit
RAM	Random-Access Memory
SEU	Single Event Upset
